# Clinical, Patient Experience and Service Outcomes of a Leisure Centre–Based NHS Musculoskeletal Physiotherapy Service: A Service Evaluation

**DOI:** 10.1002/msc.70272

**Published:** 2026-10-09

**Authors:** George McNamee, Victoria Roberts, Christopher Newton, Eilis O'Donnell, Chris Panter, Gladness Manecka, Simon Blay, Gurpreet Singh

**Affiliations:** ^1^ Department of Allied Health, School of Health and Wellbeing University of Worcester Worcester UK; ^2^ Physiotherapy Department University Hospitals Coventry and Warwickshire NHS Trust Coventry UK; ^3^ School of Health and Care Coventry University Coventry UK; ^4^ CV Life Coventry UK

**Keywords:** accessible care, Musculoskeletal, physiotherapy

## Abstract

**Background:**

Musculoskeletal (MSK) conditions place substantial burden on individuals and healthcare systems, increasing the demand for accessible, community‐based care. This service evaluation assessed an NHS MSK physiotherapy pilot delivered in a leisure centre in a socioeconomically deprived area.

**Methods:**

A retrospective evaluation of routinely collected data was undertaken for patients attending between August 2025 and January 2026. Adults referred for physiotherapy for MSK conditions received treatment in a community leisure centre. Outcomes at initial and final appointments included MSK health (MSK‐HQ), pain intensity (NPRS), physical activity (MSK‐HQ physical activity item), and health‐related quality of life (EQ‐VAS). Linear mixed‐effects models evaluated clinical outcomes. Patient experience and service utilisation data were analysed descriptively.

**Results:**

Sixty‐four patients (mean age 50.6 ± 14.8 years; 60.9% female) were included, predominantly from socioeconomically deprived areas (mean Index of Multiple Deprivation 2.4 ± 1.5). 53.2% were from ethnic minority backgrounds and 13 languages were represented. Significant improvements were observed across all outcomes: MSK‐HQ (+11.2), NPRS (−2.2), EQ‐VAS (+19.9), and physical activity (+1.8 days/week) (all *p* ≤ 0.002). Sensitivity analyses yielded similar results except for physical activity. Minimal clinically important thresholds were exceeded for MSK‐HQ, NPRS and EQ‐VAS. Patient satisfaction was high, attendance was 72.2%, and 17.2% of patients were referred to community exercise programmes.

**Conclusions:**

A leisure centre–based NHS MSK physiotherapy model was associated with improved clinical outcomes, high patient acceptability and promising service utility. The service engages a heterogeneous population and aligns with NHS priorities for community‐based care. Further comparative and economic evaluations are warranted to establish effectiveness and inform future service development.

## 1 Background

Musculoskeletal (MSK) conditions, including muscle and joint pain, account for approximately 20%–30% of General Practitioner (GP) consultations and represent a leading cause of repeat appointments (Keavy et al. 2023; Demont et al. 2019). The economic burden associated with MSK conditions is substantial, with costs to the National Health Service (NHS) estimated at £4.67 billion annually, a figure projected to rise alongside increasing prevalence and demand (Health Education England 2020). In the United Kingdom (UK), more than one third of adults live with a long‐term MSK condition, with the burden disproportionately concentrated in areas of socioeconomic deprivation (Public Health England 2020; Arthritis, 2023). MSK conditions are also a leading contributor to years lived with disability globally (Vos et al. 2020), and are associated with reduced quality of life, work absence, and high healthcare utilisation (Health and Safety Executive 2022).

In response to these challenges, there has been increasing emphasis on transforming MSK service delivery towards more sustainable and accessible models of care. The UK Government's 10 Year Health Plan (Department of Health and Social Care 2025) outlines a strategic shift from hospital‐based provision to community‐based services, aiming to improve access, reduce health inequalities, and deliver more integrated, person‐centred care. This supports NHS priorities for prevention, early intervention, and self‐management, while delivering care closer to patients' homes (Department of Health and Social Care 2025; NHS England 2019). However, traditional hospital‐based pathways may present barriers to access, particularly for individuals living in socioeconomically deprived communities, thereby perpetuating health inequalities (Cookson et al. 2016).

Physiotherapy is central to contemporary MSK care, with evidence and clinical guidelines supporting exercise therapy, education, and self‐management as first‐line interventions for common MSK conditions (such as low back and shoulder pain and knee osteoarthritis) (NICE 2020; Lin et al. 2019). In addition to improving pain and function, physiotherapy‐led care has been associated with a reduced use of low‐value interventions, including unnecessary imaging, opioid prescribing and downstream costs (Bornhöft et al. 2019; Ojha et al. 2016; Fritz et al. 2015). Despite this, access to physiotherapy services remains variable, with barriers including waiting times and lower engagement among underserved populations, suggesting that optimising how and where physiotherapy is delivered is critical to maximising its impact.

Delivering physiotherapy care within community settings may improve accessibility and engagement, particularly when co‐located within facilities that encourage health‐promoting behaviours, such as physical activity and long‐term self‐management (CSP 2024; WHO 2020). In the UK, leisure centres are community‐based public recreational facilities that provide access to health, fitness, and sports amenities, including gyms, swimming pools, fitness studios, and sports halls. These facilities may therefore provide a unique environment in which to integrate physiotherapy services with opportunities to support this model of care. Furthermore, community‐based care may help address health inequalities by reducing barriers to care, particularly in deprived populations.

Despite this, there remains limited real‐world evaluation of co‐located MSK physiotherapy services embedded within community‐based leisure facilities. This is particularly important in socioeconomically deprived areas, where innovative models may offer the greatest impact but remain underexplored. Robust evaluation of such services is therefore needed to inform future service development and commissioning decisions.

In response, University Hospitals Coventry and Warwickshire NHS Trust (UHCW), in partnership with CV Life, a Coventry‐based not‐for‐profit organisation that delivers a wide range of leisure, sport, and cultural services to promote inclusive participation, community engagement, and improved health and wellbeing, piloted an innovative model of care, Community Access to Rehabilitation and Exercise (C.A.R.E). This initiative co‐located NHS MSK physiotherapy services within a community leisure facility situated in one of Coventry's most socioeconomically deprived areas (Index of Multiple Deprivation (IMD) decile 2) (Ministry of Housing, Communities and Local Government, 2025). By integrating clinical rehabilitation with a community‐based exercise environment, the model aimed to improve access, support self‐management, and enhance patient outcomes. This service evaluation therefore aimed to assess the utility, clinical outcomes, and patient satisfaction associated with the C.A.R.E pilot.

## 2 Methods

### 2.1 Study Design

A retrospective service evaluation was conducted in line with NHS guidance for service evaluations (NHS Health Research Authority 2021). This negates the need for formal research ethics approval; however, local approval was granted in accordance with the UK Governance Arrangements for Research Ethics Committees (GAfREC) by the UHCW Research & Development Department on 27 November 2025 (Reference number SE0431).

### 2.2 Setting and Patients

The service was delivered at a community‐based leisure centre in Coventry, one day per week by two physiotherapists (1 Female, 1 Male): a Band 7 Clinical Specialist/Clinical Team Lead Physiotherapist and a Band 6 Senior Physiotherapist with a mean 8 years of clinical experience. Patients referred to UHCW's MSK physiotherapy service who resided in the local area were offered appointments at this clinic. Inclusion criteria comprised adults (≥ 18 years) with MSK pain referred to the physiotherapy service. Those who did not meet these criteria were excluded.

### 2.3 Intervention

Physiotherapy assessment and treatment interventions were delivered in accordance with the usual and best MSK physiotherapy practice (Lin et al. 2019) mirroring care delivered in the established UHCW MSK physiotherapy service. This included, but was not limited to, individualised exercise prescriptions, rehabilitation programmes, and education to support self‐management. Patients were also provided with advice on lifestyle modification and physical activity to facilitate long‐term condition management.

Co‐locating the service within a leisure centre enabled signposting to on‐site exercise and wellbeing programmes, supporting self‐management via engagement with physical activity beyond the physiotherapy intervention (CV Life 2026).

### 2.4 Data Collection

A retrospective evaluation of routinely collected data within this pilot service was conducted for the pilot period between August 2025 and January 2026.

#### 2.4.1 Patient Demographics and Characteristics

Patient demographic and characteristic data were collected at the initial appointment included age, sex, ethnicity, and main spoken language; distance lived from the leisure centre and IMD level of home address (Ministry of Housing and Communities and Local Government 2025); height, weight and body mass index (BMI); number and type of health‐related comorbidities; number of pain sites; MSK condition referred for; and work status. For patients in employment, a self‐reported record of periods and days of MSK‐related work absence was recorded for the 13 weeks prior to their initial appointment.

#### 2.4.2 Clinical Outcomes

Self‐reported clinical outcome measures were collected at the initial and final appointments with the physiotherapy service. These measures included MSK health status (Musculoskeletal Health Questionnaire (MSK‐HQ) (0‐56 scale, with higher scores indicating better MSK health)) (Hill et al. 2016); average days of physical activity during the last week (MSK‐HQ physical activity item (0‐7 scale, with higher scores indicating greater days physically active)) (Hill et al. 2016); average pain intensity during the last week (Numerical Pain Rating Scale (NPRS) (0‐10 scale, where higher scores indicate greater pain intensity)) (Hawker et al. 2011); and a measure of self‐rated overall health (EuroQol Visual Analogue Scale (EQ‐VAS) (0‐100 scale, with higher scores indicating greater overall health)) (Herdman et al. 2011). These measures were selected based on their established psychometric properties, including good validity, reliability, and responsiveness in MSK populations, and their widespread use in both clinical practice and research settings.

#### 2.4.3 Patient Satisfaction and Experience

A structured questionnaire (see Suppoting Information S1: File 1) was devised to capture patients' experience of and satisfaction with accessibility, location, perceived quality of care, and acceptability of the leisure centre‐based MSK service (C.A.R.E). The questionnaire was constructed by the project team for the purposes of this service evaluation. Three patients from the UHCW MSK physiotherapy service reviewed the questionnaire and provided feedback on the clarity and appropriateness of the questions, which informed minor refinements prior to use.

#### 2.4.4 Service Utility

Service utilisation metrics were routinely collected to evaluate the uptake and operational performance of the service. These included attendance rates (including completed appointments and non‐attendance), and the proportion of patients referred to CV Life's exercise and wellbeing programmes.

### 2.5 Data Analysis

Patients' demographics and characteristics, patient satisfaction and experience, and service utility data were reported descriptively as means, standard deviations (SD), totals and percentages as appropriate. The number of patients lost to follow‐up was recorded for documented reasons.

Statistical analysis was conducted in SPSS Statistics 26 with changes in self‐reported clinical outcomes between the initial and final appointments being examined using linear mixed‐effects models (Gueorguieva and Krystal 2004). The initial and final appointments timepoints were specified as a fixed effect, with each participant included as a random intercept for account for repeated measurements. Models were estimated using restricted maximum likelihood (REML) with a variance‐component covariance structure. All available outcome data were included in the models under the assumption that missing data were missing at random. Estimated marginal means (SE), mean changes with 95% confidence intervals (CIs), and *p*‐values were reported, with statistical significance set at *p* < 0.05. Mean change scores were compared to accepted minimal clinically important differences (MCID) of 5.5 for the MSK‐HQ (Scott et al. 2020; Price et al. 2019) and 2 points for the NPRS (Salaffi et al. 2004; Farrar et al. 2001). Given the absence of an established MCID for the EQ‐VAS in heterogeneous MSK populations, a 13‐point improvement was used as a pragmatic threshold for clinically meaningful change. This threshold was informed by the mean MCID estimates reported across MSK populations in a recent systematic review (Cheng et al. 2024).

Complete‐case sensitivity analyses were subsequently conducted using paired‐sample *t*‐tests among participants with both initial and final outcome measurements to examine the influence of missing follow‐up data on the results.

## 3 Results

Between 01 August 2025 and 31 January 2026, 77 new patients were offered an initial appointment with the pilot service, of which 64 (83.1%) attended and provided baseline measures. 27 (42.2%) patients failed to attend a subsequent appointment and made no further contact with the physiotherapy service so were classed as lost to follow‐up. 4 (6.3%) patients rescheduled their final appointment during the pilot period and were followed up within the standard UHCW physiotherapy service once the pilot finished. Repeat measures and questionnaires were collected for the remaining 51.6% (*n* = 33) of these patients (see Figure 1).

**FIGURE 1:**
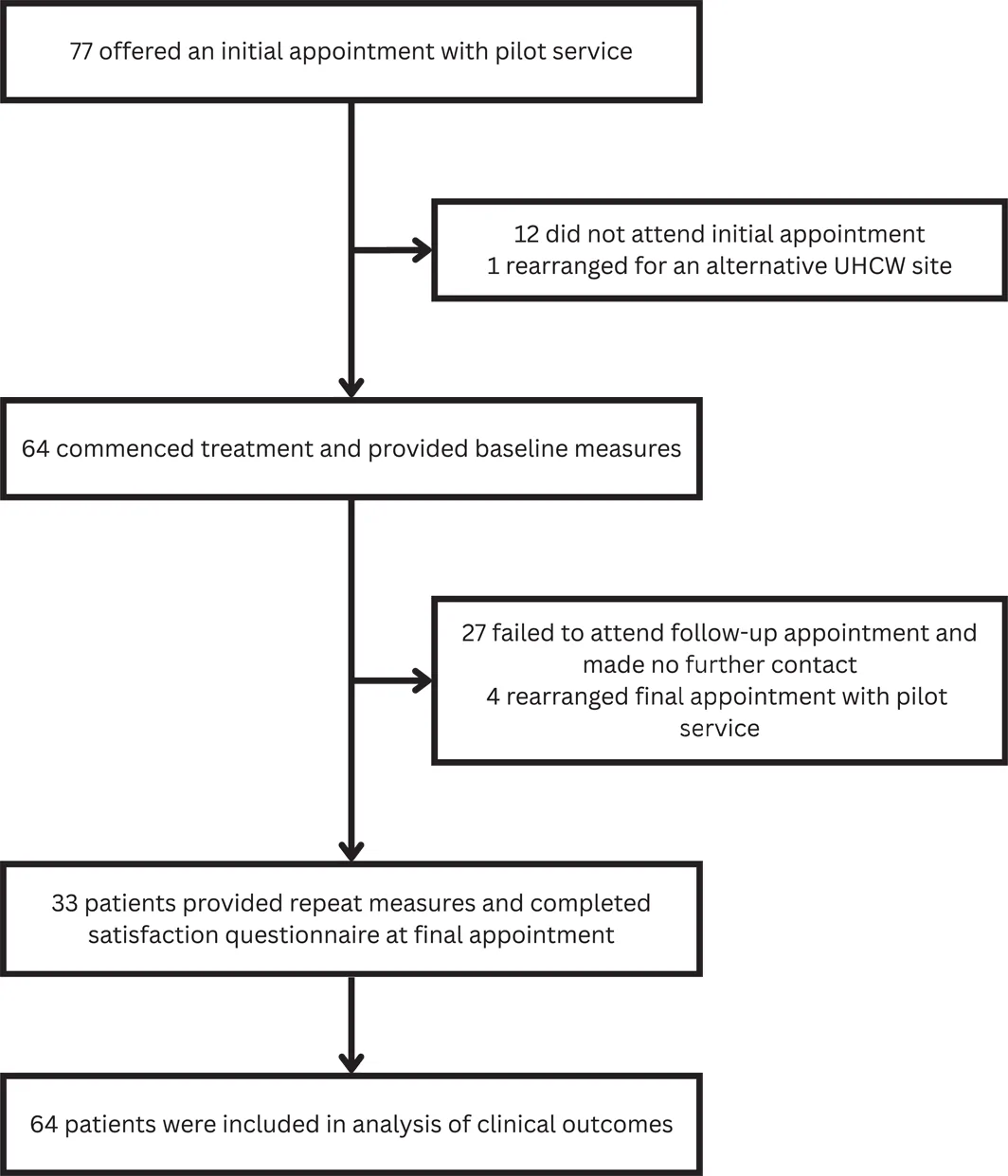
Patient flow diagram.

### 3.1 Patient Demographics and Characteristics

Patients represented a heterogeneous cohort (see Table 1). The service engaged a diverse population, with over half (53.2%) of patients identifying as belonging to ethnic minority communities and 13 different native languages spoken across the cohort. On average, patients lived 1.5 miles ( ± 1.5) from the leisure centre and predominantly resided in areas of high socioeconomic deprivation (mean IMD level of 2.4 ( ± 1.5)). Reasons for referral into the UHCW MSK physiotherapy service covered an array of MSK conditions; despite only 3 (4.7%) referrals for multi‐site pain, patients reported a mean of 3.0 ( ± 2.7) pain sites.

**TABLE 1:** Patients' demographics and characteristics.

		Patients (*n* = 64)
Age, years		50.6 (± 14.8)
Sex	Female	39 (60.9%)
	Male	25 (39.1%)
Ethnicity	White	30 (46.9%)
	Asian	25 (39.1%)
	Black	9 (14.1%)
Main spoken language	English	47 (74.6%)
	Other (spoken by *n* = 1 patient each)	10 (15.9%)
	Punjabi	5 (7.9%)
	Urdu	2 (3.2%)
Distance from home address to leisure centre, miles		1.5 (± 1.5)
IMD level of home address		2.4 (± 1.5)
BMI, kg/m^2^		30.8 (± 6.6)
Number of comorbidities		1.9 (± 1.9)
Comorbidity, type	Musculoskeletal	28 (43.8%)
	Cardiac	24 (37.5%)
	Metabolic	18 (28.1%)
	Mental health	16 (25.0%)
Number of pain sites		3.0 (± 2.7)
MSK condition referred for	Low back pain	24 (37.5%)
	Knee pain	13 (20.3%)
	Neck pain	8 (12.5%)
	Foot/Ankle pain	7 (10.9%)
	Shoulder pain	6 (9.4%)
	Multiple site pain	3 (4.7%)
	Wrist/Hand pain	2 (3.1%)
	Hip pain	1 (1.6%)
Work status	Employed	27 (42.2%)
	Unemployed	25 (39.1%)
	Retired	12 (18.8%)
MSK‐related work absence for employed patients		5 (18.6%)
Days absent from work due to MSK, last 13 weeks		15.8 (± 8.9)
Musculoskeletal health status, MSK‐HQ score		22.2 (± 9.9)
Days physically active, MSK‐HQ PA score		2.4 (± 2.4)
Pain: Single item, average past week, NPRS score		7.2 (± 1.7)
Overall health score, EQ‐VAS score		49.3 (± 21.4)

*Note:* Data are *n* (%) or mean (± SD). Other languages spoken by *n* = 1 patient = Arabic, Bengali, Croatian, French, Kurdish, Latvian, Polish, Swahili, Tamil, Twi.

Abbreviations: BMI, Body mass index; EQ‐VAS, EuroQol visual analogue scale; IMD, Index of Multiple Deprivation; MSK, Musculoskeletal; MSK‐HQ, Musculoskeletal Health Questionnaire; NPRS, Numerical Pain Rating Scale; PA, Physical activity.

### 3.2 Clinical Outcomes

Statistically significant improvements from baseline were observed post‐treatment across all self‐reported clinical outcomes for this cohort (see Table 2). Where established MCID thresholds were available, improvements also met or exceeded the criteria for meaningful clinical change. Patients' self‐reported musculoskeletal health status change (MSK‐HQ) exceeded the MCID (≥ 5 points), with mean improvements of 11.2 points at the final appointment (95% CI 6.0 to 16.5, F(1, 39.1) 19.0, *p* < 0.001). Pain intensity (NPRS) also exceeded MCID improvements (≥ 2 points), with reductions of 2.2 points (95% CI −3.2 to −1.3, F(1, 39.7) 21.1, *p* < 0.001). Patients' overall health rating exceeded the MCID (≥ 13 points) with improvements of 19.9 (95% CI 10.9 to 29.0, F(1, 53.5) 19.4, *p* < 0.001). Patients' levels of physical activity significantly increased after attending the service with a mean increase of 1.8 days active per week (95% CI 0.7 to 2.9, F(1, 49.3) 10.3, *p* = 0.002).

**TABLE 2:** Self‐reported clinical outcomes.

	Timepoint	Mean (SE)	Mean difference from baseline (SE)	95% CI		*p* value
				Lower	Upper	
Musculoskeletal health status: MSK‐HQ score	Initial	22.2 (1.9)				
	Final	33.4 (2.7)	11.2 (2.6)	6.0	16.5	< 0.001
Physical activity levels, days: MSK‐HQ PA score	Initial	2.4 (1.7)				
	Final	4.2 (1.8)	1.8 (0.6)	0.7	2.9	0.002
Pain: Single item, average past week, NPRS score	Initial	7.2 (1.3)				
	Final	5.0 (1.3)	−2.2 (0.5)	−3.2	−1.3	< 0.001
Overall health rating, EQ‐VAS score	Initial	48.7 (18.7)				
	Final	68.6 (18.8)	19.9 (4.5)	10.9	29.0	< 0.001

Abbreviations: EQ‐VAS, EuroQol Visual Analogue Scale; MSK‐HQ, Musculoskeletal Health Questionnaire; NPRS, Numerical Pain Rating Scale; PA, Physical activity.

Complete‐case sensitivity analyses (see Suppoting Information S1: File 2) produced similar estimates to the primary mixed‐model analyses for the MSK‐HQ, NPRS and EQ‐VAS. For the MSK‐HQ physical activity item, the complete‐case analysis produced a smaller estimated improvement than the primary mixed‐model analysis that did not exceed statistical significance (0.8 days, 95% CI −0.1 to 1.7, *p* = 0.079).

### 3.3 Service Satisfaction

All patients (*n* = 33, 100.0%) responded positively when asked if they would recommend the leisure centre‐based MSK service to others with similar problems, with 30 (90.9%) patients “definitely” recommending the service. All patients (*n* = 33, 100.0%) reported positively on the convenience of the leisure centre location for their appointments, with 84.8% (*n* = 28) rating access as “very easy”. Furthermore, 81.8% (*n* = 27) of participants felt the non‐hospital location was “definitely” beneficial for their treatment, with the remaining 18.2% (*n* = 6) feeling the location was “somewhat” beneficial for their MSK care.

Most patients (*n* = 31, 93.9%) reported increased confidence to exercise following their care at the leisure centre, with 75.8% (*n* = 25) of patients reporting this was facilitated by being in the leisure centre environment All patients (*n* = 33, 100.0%) reported that they intended to continue exercising regularly for the long‐term (see Figure 2).

**FIGURE 2:**
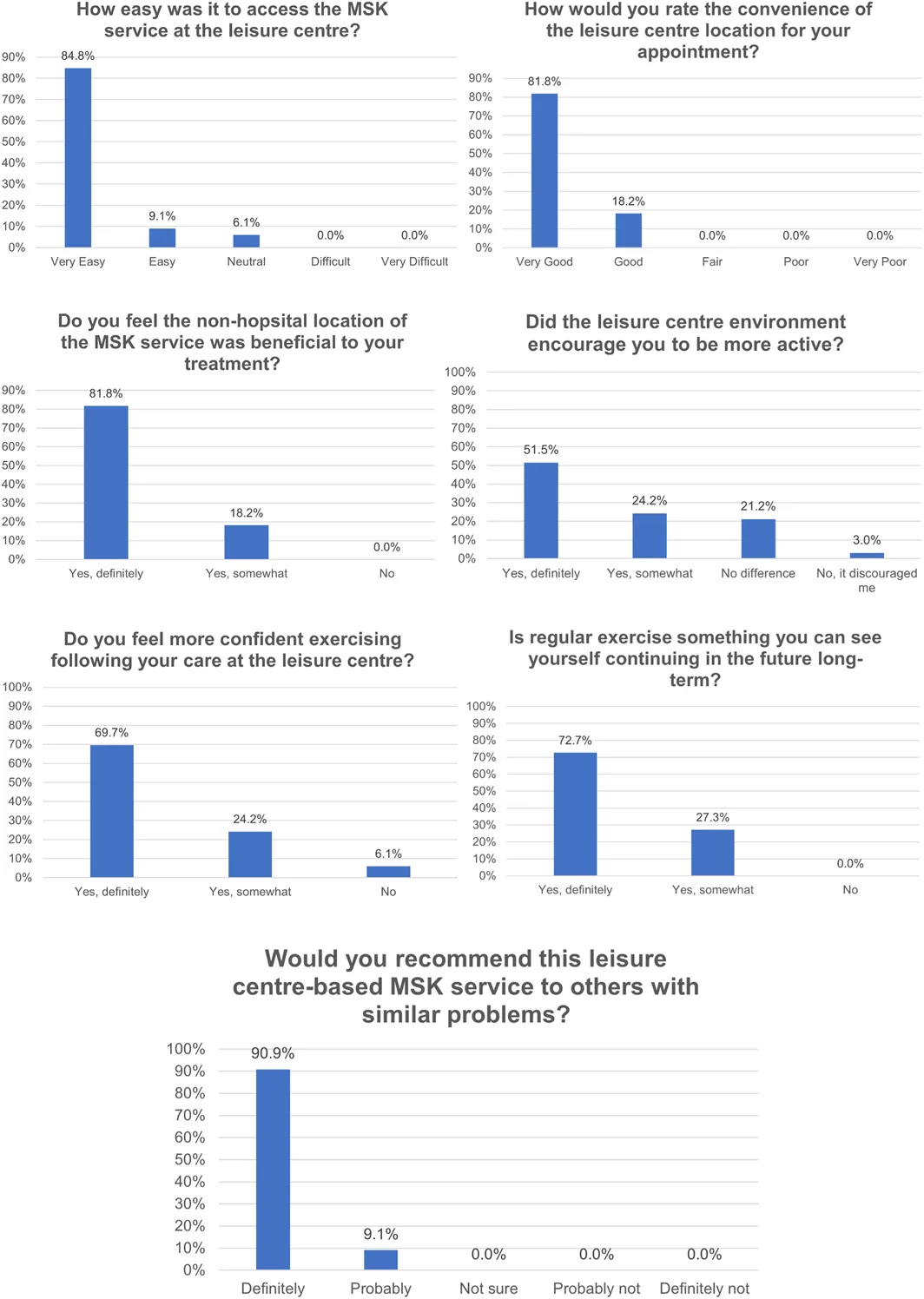
Responses to the patient satisfaction and experience questionnaire.

### 3.4 Service Utility

Sixty‐four patients (83.1%) patients attended their initial MSK appointment at the leisure centre. In total, 216 appointments were booked over the 6‐month pilot with a 72.2% (*n* = 156) attendance rate. Patients rescheduled 9.7% (*n* = 21) of their appointments, with two‐thirds of these being for follow‐up (FU) appointments (*n* = 14, 66.6%). 39 (18.1%) appointments were not attended by patients, with the majority of these being for FU appointments (*n* = 27, 69.2%).

On average, patients attended 2.7 ( ± 1.5) appointments, although this increased to 3.8 ( ± 2.0) for those that completed treatment within the pilot. Nearly half of the patients (*n* = 29, 45.3%) completed their treatment within the pilot period, with a further 8 (12.5%) continuing their care within UHCW located physiotherapy services once the pilot finished. The remaining 27 (42.2%) patients were discharged following a period of no contact with the service.

Eleven (17.2%) patients who received physiotherapy care during the pilot were referred into CV Life's exercise and wellbeing programmes (see Table 3).

**TABLE 3:** Service utility outcomes.

Appointment booked		216
Appointments attended		156 (72.2%)
Appointments rescheduled		21 (9.7%)
	Follow‐up	14 (66.6%)
	Initial	7 (33.3%)
Appointments not attended		39 (18.1%)
	Follow‐up	27 (69.2%)
	Initial	12 (30.8%)
End of pilot destination	Treatment completed in pilot	29 (45.3%)
	Discharged following no contact	27 (42.2%)
	Remained on treatment	8 (12.5%)
Number of sessions	Overall	2.7 ( ± 1.5)
	Completed treatment	3.8 ( ± 2.0)
Pilot referrals into CV life		11 (17.2%)
UHCW physiotherapy referrals into CV life^a^		15 (2.4%)

^a^ Figures were captured from a snapshot of 638 patients seen by 16 clinicians over 1 week within UHCW located MSK physiotherapy services.

## 4 Discussion

This service evaluation aimed to assess the clinical outcomes, patient experience, and service utility of this novel leisure centre–based NHS MSK physiotherapy service. These findings suggest that co‐locating MSK physiotherapy within a leisure centre is associated with high patient satisfaction and improvements in self‐reported pain, musculoskeletal health, physical activity, and health‐related quality of life. Statistically significant improvements were observed across all self‐reported outcomes, while improvements exceeded established MCID thresholds for those outcomes where such thresholds are available. Collectively, these findings suggest that this model may offer meaningful benefits for individuals seeking physiotherapy care for MSK conditions. Furthermore, the service aligns with growing calls to deliver accessible, person‐centred MSK care within community settings and to address health inequalities through locally available healthcare provision (Department of Health and Social Care 2025; NHS England 2019).

The clinical outcomes observed compare favourably with other community‐based MSK initiatives. For example, a large‐scale community MSK Hub programme delivered to over 1600 patients across 94 sites showed that only 30%–40% of patients achieved MCID thresholds (ukactive Research Institute 2024). While direct comparisons should be interpreted cautiously due to differences in interventions and evaluation methods, the present service evaluation reached a more heterogeneous population than the MSK Hub programme (ukactive Research Institute 2024) and also demonstrated statistically significant improvements across all outcomes measured, with clinically meaningful improvements observed for outcomes with established MCID thresholds. These findings suggest that co‐located physiotherapist‐led MSK care in community‐based leisure settings can be delivered successfully and warrant further evaluation using comparative study designs.

The leisure centre location of this pilot helped facilitate physical activity as a core component of rehabilitation. Firstly, the post‐treatment increase in days physically active per week (+1.8 days, *p* = 0.002) represents a notable improvement from baseline measures. Secondly, suggestions that co‐location of MSK physiotherapy services within leisure centres can facilitate positive changes in physical activity behaviours can be supported given that the leisure centre environment encouraged patients to be more active (75.8%), and that patients felt more confident to exercise (93.9%), and to maintain this for the long‐term (100.0%). This is consistent with the literature emphasising the importance of environmental context and opportunity in supporting sustained behaviour change (Michie et al. 2011). The increased confidence to engage in exercise reported by patients may also contribute to longer‐term self‐management and reduced reliance on healthcare services, aligning with policy priorities to shift care closer to home and emphasise prevention (Department of Health and Social Care 2025; NHS England 2019).

A novel finding of this service evaluation was that 17.2% of patients receiving care within this pilot were referred onward to CV Life's exercise and wellbeing programmes compared to 2.4% of patients receiving care in UHCW located services during a one‐week service snapshot. While these figures are not directly comparable and do not permit conclusions regarding the effectiveness of the service model, they suggest that co‐location may facilitate opportunities for onward referral to leisure‐centre based health and wellbeing provision. This could be explained by 75.8% of patients reporting that the leisure centre environment encouraged them to be more active and all patients reporting that they intended to continue exercising in the long‐term, with the community‐based programmes offered by CV Life providing a valuable pathway to facilitate this. Furthermore, the co‐location of the MSK physiotherapy service in a CV Life leisure centre presented the opportunity for a smoother transition between services. Despite the increased proportion of patients being referred into these programmes, which is important for self‐management, long‐term health and behaviour change, the extent to which patients remain engaged with these services was not measured. In the future, understanding facilitators and barriers to onward physical activity engagement will be important in optimising the integration of clinical and community pathways.

### 4.1 Strengths and Limitations

A notable strength of this pilot was the diversity of the patient cohort, which included a high proportion of individuals from ethnic minority communities (*n* = 34, 53.1%), those living in areas of socioeconomic deprivation (mean IMD 2.4 ± 1.5), and individuals with multiple comorbidities (mean 1.9 ± 1.9). These populations are commonly underrepresented in MSK research and are known to experience disparities in access to, and outcomes from, healthcare services (McNamee et al. 2025; Pardhan et al. 2025; Barnett et al. 2012). The ability of this pilot to engage such groups suggests that co‐locating services within familiar, community‐based environments could reduce barriers to access and improve equity. The relatively short mean travel distance (1.5 miles ± 1.5) further supports the importance of geographical accessibility, which has been identified as a key determinant of healthcare utilisation (Kelly et al. 2016).

Service utilisation metrics highlight both the strengths and limitations of this service evaluation. The number of missed appointments (DNA) (*n* = 39, 18.1%) is notable and reflects a persistent challenge across health services (Philpott‐Morgan et al. 2021). Whilst the DNA rates reported in this service evaluation are higher than those reported by other outpatient services at UHCW (9.0%) (NHS 2026), the physiotherapy service is not specifically recorded in this dataset and our finding of 18.1% is not too dissimilar to DNA rates (15.2%) in other Primary Care services (Parsons et al. 2021). Given that the pilot predominantly served patients living in areas of higher social deprivation, the observed DNA rate may be consistent with previous evidence demonstrating an association between socioeconomic deprivation and increased non‐attendance, with figures rising to 20% for this group (NHS England 2023; Campbell et al. 2015). It is also noteworthy that the delivery of this pilot was restricted to a single clinic day per week, which may have influenced attrition due to a lack of flexibility and appointment availability to accommodate patients' schedules. Whilst there is growing evidence seeking to gain an understanding of missed appointments in healthcare (Sung et al. 2024; NHS England 2023; Philpott‐Morgan et al. 2021), further work is required to understand the reasons for non‐attendance within the C.A.R.E pilot and similar models.

It is important to acknowledge that the retrospective design and absence of a comparator group are methodical limitations of this service evaluation. The number of patients lost to follow‐up was substantial (*n* = 27, 42.2%) and may introduce attrition bias. Furthermore, the pilot operated at a smaller capacity to UHCW located physiotherapy services, with two experienced physiotherapists delivering care in a single clinic day per week, which may limit the generalisability of findings to larger‐scale implementation. Despite these limitations, the findings offer important insights into the feasibility, acceptability, and early clinical impact of this model of care.

## 5 Conclusion

This service evaluation suggests that a co‐located, leisure centre‐based MSK physiotherapy service is feasible, acceptable to patients, and associated with improvements in self‐reported MSK health, pain, and health‐related quality of life, with increased physical activity in the primary analysis. The service engaged a heterogeneous population and was well received by patients, many of whom reported that the non‐hospital setting supported positive health and lifestyle behaviours. These findings are broadly consistent with contemporary approaches to MSK service transformation, which emphasises community‐based delivery, self‐management, and multidisciplinary integration. Further research is needed to evaluate this model at scale, including comparative effectiveness studies and economic evaluations, to inform commissioning and service design decisions.

## Author Contributions

The concept for this pilot service was conceived by G.S., C.N., C.P. and E.O’D., G.Mc., G.S. and C.N. developed the concept of this service evaluation. The service evaluation was managed by G.Mc. and V.R. Data was analysed and the first draft of the manuscript was constructed by G.Mc. All authors critically revised the manuscript for important intellectual content and met the International Committee of Medical Journal Editors definition of authorship.

## Funding

The authors have nothing to report.

## Ethics Statement

A retrospective service evaluation was conducted in line with NHS guidance for service evaluations (NHS Health Research Authority, 2021). This negates the need for formal research ethics approval; however, local approval was granted in accordance with the UK Governance Arrangements for Research Ethics Committees (GAfREC) by the UHCW Research & Development Department on 27 November 2025 (Reference number SE0431).

## Conflicts of Interest

G.Mc., G.S. and C.N. receive fees for delivering workshops on person‐centred care. V.R., E.O’D., C.P., G.M. and S.B. have no declarations of interest.

## Supporting information


Supporting Information S1

## Data Availability

The data that support the findings of this study are available on request from the corresponding author. The data are not publicly available due to privacy or ethical restrictions.
