# Supplementary material for: Clinical, Patient Experience and Service Outcomes of a Leisure Centre–Based NHS Musculoskeletal Physiotherapy Service: A Service Evaluation
Source: Musculoskeletal Care. 2026 Oct 9;24(4):e70272. doi: 10.1002/msc.70272 (PMC13650786; doi:10.1002/msc.70272)
Supplement: Supplementary file 1 [file MSC-24-e70272-s001.docx]

**Supplementary File 1: Patient satisfaction and experience questionnaire**

1) How easy was it to access the MSK service at the leisure centre?

Easy

Very

Easy

Neutral

Difficult

Very Difficult

2) How would you rate the convenience of the leisure centre location and hours for your appointments?

Good

Very Good

Fair

Poor

Very Poor

3a) Do you feel that the non-hospital location of the MSK service was beneficial to your treatment in any way?

Yes, definitely

Yes, somewhat

No

3b) Please use this space to expand on the answer above, if you wish:

4) Did the leisure centre environment (gym, pool, classes) encourage you to be more active during or after your treatment?

No difference

Yes, somewhat

No, it discouraged me

Yes, definitely

5) Since your first appointment with the MSK service, how often are you engaging with physical activity or exercise?

Less than once a week

1-2 times a week

Not at all

3 times or more a week

6) Do you feel more confident exercising following your care at the leisure centre?

Yes, definitely

Yes, somewhat

No

7) Is regular exercise now something you can see yourself continuing in the future long-term?

Yes, definitely

Yes, somewhat

No

8) Would you recommend this leisure centre-based MSK service to others with similar problems?

Probably

Definitely

Not sure

Probably not

Definitely not

**Thank you for taking the time to answer these questions; your experience is important to us.**

**Supplementary File 2: Complete-case sensitivity analyses**

The complete-case analyses included participants with both initial and final outcome measurements using a paired-samples t-test.

|  | **Timepoint** | **Mean (±SD)** | **Mean difference from baseline** | **95% CI** | | ***p* value** | **Cohen’s *d*** |
| --- | --- | --- | --- | --- | --- | --- | --- |
|  |  |  |  | **Lower** | **Upper** |  |  |
| Musculoskeletal health status: MSK-HQ score | Initial | 21.7 (9.56) |  |  |  |  |  |
|  | Final | 33.4 (11.3) | 11.7 | 7.1 | 16.4 | <0.001 | 1.04 |
| Physical activity levels, days: MSK-HQ PA score | Initial | 3.4 (2.6) |  |  |  |  |  |
|  | Final | 4.2 (2.3) | 0.8 | -0.1 | 1.7 | 0.079 | 0.376 |
| Pain: single item, average past week, NPRS score | Initial | 7.5 (1.3) |  |  |  |  |  |
|  | Final | 4.8 (2.1) | -2.7 | -3.8 | -1.7 | <0.001 | 1.07 |
| Overall health rating, EQ-VAS score | Initial | 52.3 (21.2) |  |  |  |  |  |
|  | Final | 68.6 (17.9) | 15.7 | 5.8 | 25.6 | 0.003 | 0.65 |
| MSK-HQ = Musculoskeletal Health Questionnaire. PA = Physical activity. NPRS = Numerical Pain Rating Scale. EQ-VAS = EuroQol Visual Analogue Scale. | | | | | | | |

***Table 3:* Complete-case sensitivity analysis of self-reported clinical outcomes.**
